# Prognostic Value of the Preoperative HALP Score for Survival in Non-Malignant Liver Transplant Recipients

**DOI:** 10.3390/jcm15155970

**Published:** 2026-07-30

**Authors:** Muzaffer Atlı, Halil Şahin, Lütfi Soylu, Sedat Karademir

**Affiliations:** 1 Department of General Surgery, Istanbul Aydın University, 34295 Istanbul, Turkey; 2 Department of Gastroenterology and Hepatology, Istanbul Aydın University, 34295 Istanbul, Turkey; halilshn33@gmail.com; 3 Department of General Surgery, Ankara Guven Hospital, 06540 Ankara, Turkey; lutfisoylu@hotmail.com; 4 Department of General Surgery, Anadolu Medical Center, 41400 Kocaeli, Turkey; sedatkarademir@gmail.com

**Keywords:** liver transplantation, HALP score, immunonutrition, prognosis, survival, nutritional status

## Abstract

**Background/Objectives:** The HALP score is a simple immunonutritional index calculated from routine preoperative hemoglobin, albumin, lymphocyte, and platelet values. We evaluated its association with mortality after liver transplantation for non-malignant indications and its prognostic contribution when considered with the Model for End-stage Liver Disease (MELD) score. **Methods:** We retrospectively analyzed 194 adults who underwent liver transplantation for non-malignant disease at a single center between January 2015 and January 2025. The primary outcome was all-cause post-transplant death. The cohort-derived HALP threshold was used for exploratory Kaplan–Meier and Cox analyses, while continuous HALP was examined in sensitivity analyses. Censoring-adjusted cumulative/dynamic ROC analyses evaluated discrimination at 30 days, 90 days, 1 year, and 3 years. **Results:** Over a median observed follow-up of 1108 days, 43 recipients (22.2%) died. Non-survivors had lower HALP values than survivors [0.52 (0.39–0.67) vs. 0.77 (0.54–1.04); *p* < 0.001]. HALP had the highest observed conventional AUC among the evaluated markers for overall mortality (AUC 0.735; 95% CI 0.657–0.814); pairwise AUC differences were not formally tested. The exploratory threshold was 0.6786. Mortality occurred in 36.3% of the low-HALP group and 9.7% of the high-HALP group. After adjustment for MELD and albumin, high versus low HALP remained associated with lower mortality (HR 0.239; 95% CI 0.116–0.493; *p* < 0.001). HALP also showed consistent time-dependent discrimination across the four evaluated horizons (AUC 0.713–0.738). **Conclusions:** Lower preoperative HALP was associated with higher post-transplant mortality in this non-malignant liver transplant cohort. HALP may provide complementary risk information, but the cohort-derived threshold requires independent external validation before clinical use.

## 1. Introduction

Liver transplantation (LT) remains widely accepted as the standard and curative therapy in end-stage liver disease [1]. Although advances in operative methods, perioperative care, and immunosuppression have markedly improved short-term survival, only limited improvement has been achieved in long-term outcomes in recent years [2,3]. Despite the increasing volume of liver transplantation, the continued clinical burden of long-term mortality highlights the need to support existing risk prediction models with complementary biomarkers [3,4].

Post-transplant survival is shaped not just by surgical success but equally by the recipient’s biological reserve and overall resilience. End-stage liver disease is not confined to the liver; it is a systemic condition in which protein-energy malnutrition, sarcopenia, cirrhosis-associated immune dysfunction, and cytopenias arising from hypersplenism and impaired marrow reserve accumulate and progressively erode physiological reserve [5,6]. Large cohort studies have shown that recipient-related factors, such as age, metabolic status, and systemic inflammation, significantly affect survival after transplantation [7,8]. The Model for End-stage Liver Disease (MELD) score is widely used to predict short-term mortality and guide organ allocation; however, it is based on creatinine, bilirubin, and INR and primarily reflects hepatorenal dysfunction. Therefore, it does not fully capture the biological reserve of the recipient [9]. MELD was derived to predict short-term waiting-list mortality and contains no measure of nutritional status, immune competence, or hematological reserve; two recipients with an identical MELD score may therefore differ substantially in the biological reserve with which they enter transplantation [10]. In contrast, frailty, sarcopenia, and malnutrition represent immunonutritional reserve independently of MELD and may have an additional determining role in transplant outcomes [11,12].

However, the tools that capture this reserve are not easily deployed in routine pretransplant practice. Frailty indices require dedicated functional testing, and sarcopenia assessment depends on cross-sectional imaging and standardized software, so neither is uniformly available at the time of listing or of transplantation [13]. There is therefore an unmet need for objective, inexpensive, and reproducible markers that can be derived from laboratory tests already obtained before transplantation and that complement MELD by reflecting the immunonutritional reserve of the recipient.

The hemoglobin, albumin, lymphocyte, and platelet (HALP) score is a composite immunonutritional index obtained as the product of hemoglobin, albumin, and lymphocyte values divided by the platelet count. It reflects systemic inflammation, immune competence, and nutritional reserve together [14]. Because it is readily obtained from standard laboratory tests, HALP is an applicable, objective, and low-cost marker in clinical practice. Its components may reflect chronic disease burden and oxygen-carrying capacity through hemoglobin, hepatic synthesis and nutritional reserve through albumin, cellular immunity through lymphocytes, and thrombo-inflammatory activity through platelets. First described in gastric cancer [15], this score has subsequently shown significant associations with clinical outcomes in numerous solid malignancies and various non-oncological diseases [16,17,18,19,20,21,22,23]. Meta-analytic data indicate that low preoperative HALP scores are associated with worse overall survival [14], while population-based analyses raise the possibility that HALP functions as a general prognostic indicator of systemic biological vulnerability [24]. Importantly, the biological meaning of a low HALP score is not necessarily the same across settings. In oncological cohorts its components are influenced by tumor burden and cancer-related systemic inflammation, whereas in advanced non-malignant liver disease the same components are driven mainly by impaired hepatic synthesis, portal hypertension with hypersplenism, and chronic immune dysfunction.

In liver-related diseases, the HALP score has been investigated in both malignant and non-malignant conditions. Low preoperative HALP values have been reported to be associated with worse outcomes after curative resection for hepatobiliary malignancies [25,26,27,28], while in chronic liver diseases, low HALP scores have been linked to more advanced disease stage and increased mortality risk [29,30]. Data also suggest that the preoperative HALP score may provide additional prognostic information in transplantation performed for hepatocellular carcinoma [31,32]. However, because a substantial proportion of the available evidence comes from malignancy-related cohorts, uncertainty remains regarding the generalizability of the HALP score to transplant populations independent of tumor burden, tumor biology, recurrence risk, and cancer-related systemic inflammation. Evidence in recipients transplanted for non-malignant indications, in whom pretransplant risk is determined by the severity of chronic liver disease rather than by tumor biology, therefore remains limited, and it is in precisely this population that a marker complementing MELD would be most useful.

Therefore, the present study evaluated the prognostic association of the preoperative HALP score with post-transplant survival in adults undergoing liver transplantation for non-malignant liver diseases and examined whether HALP provided additional prognostic information when considered with the MELD score.

## 2. Materials and Methods

### 2.1. Study Design

We performed a retrospective, single-center, observational cohort study of adults who underwent liver transplantation at the Organ Transplantation Department of Ankara Güven Hospital between January 2015 and January 2025. Reporting followed the STROBE guideline for observational research. We hypothesized that the pretransplant HALP score would relate to all-cause post-transplant mortality in patients transplanted for non-malignant indications. HALP was evaluated both as a continuous variable and as a ROC-derived categorical variable. For clinical interpretability, the low- and high-HALP groups were used for Kaplan–Meier analysis and the main clinical Cox model, while continuous HALP was examined in sensitivity analyses.

### 2.2. Study Population and Patient Selection

We assessed 252 adult recipients aged 18 years or older who underwent liver transplantation during the study period. An isolated liver transplantation was defined as liver-only transplantation without simultaneous transplantation of another organ. We excluded 47 recipients transplanted for hepatocellular carcinoma, 10 recipients with missing laboratory data required to calculate HALP, and 1 intraoperative death, leaving 194 recipients in the final complete-case cohort. For recipients who underwent retransplantation during the study period, only the first transplantation was included as the index procedure.

### 2.3. Data Sources and Collected Variables

After ethics-committee approval, anonymized data were analyzed using a predefined standardized form based on the institutional electronic medical record, transplant follow-up files, and laboratory information system. Demographics covered age and sex. Clinical fields covered the primary liver-disease etiology, donor type (living or deceased), diabetes mellitus, hypertension, and coronary artery disease. Disease severity was indexed by the pretransplant MELD score.

Recorded preoperative laboratory values were hemoglobin (g/dL), serum albumin (g/dL), absolute lymphocyte count (/µL), platelet count (/µL), aspartate aminotransferase (AST, U/L), and alanine aminotransferase (ALT, U/L). These preoperative values were obtained on the day before transplantation in every recipient, providing a standardized and uniform sampling time point across the cohort. The De Ritis ratio was defined as AST/ALT.

### 2.4. Calculation of the HALP Score

HALP was computed from the pretransplant laboratory values with the following formula:

HALP = (hemoglobin × serum albumin × absolute lymphocyte count)/platelet count(1)

Hemoglobin and serum albumin were used in g/dL, and lymphocyte and platelet counts were used as /µL; the resulting HALP values were interpreted within the same unit system. HALP was evaluated both continuously and categorically. ROC analysis was performed to assess its discriminative performance for mortality, and low and high HALP groups were formed using the threshold determined by the Youden index. This threshold was considered exploratory and was not intended to represent a universally applicable clinical cut-off.

### 2.5. Endpoints and Follow-Up Definitions

The index date was the day of transplantation. All-cause post-transplant death was the primary endpoint. Overall survival, in days, spanned transplantation to death or the last clinical contact, and patients alive at last contact were censored. Data were locked in January 2026.

A secondary composite graft-related endpoint was defined as retransplantation for graft failure or death occurring with graft failure. Because graft-related events almost completely overlapped with death in this cohort, this endpoint was interpreted descriptively rather than as death-censored graft survival. Accordingly, it is referred to throughout as a secondary composite descriptive endpoint, and it is not presented as an independent graft-failure outcome.

### 2.6. Missing Data

Among the 194 recipients included in the analysis, there were no missing data for HALP components, MELD score, AST, ALT, survival time, or survival status. A complete-case analysis was therefore performed without imputation.

### 2.7. Statistical Analysis

Analyses used IBM SPSS Statistics v27.0. Bootstrap internal validation of the optimism-corrected C-index and Firth penalized Cox regression were run separately in R. Continuous variables were checked for normality with the Shapiro–Wilk test; as continuous variables showed non-normal distributions, they were summarized as median and interquartile range (IQR) and compared with the Mann–Whitney U test. Categorical data appear as counts and percentages, compared with the Pearson chi-square test or, where expected counts were low, Fisher’s exact test.

Conventional ROC analysis was used to evaluate discrimination for overall mortality, with AUCs and 95% confidence intervals reported. The marker direction was specified so that higher risk scores consistently indicated higher mortality risk; HALP was therefore entered with reversed direction, whereas MELD and the De Ritis ratio were entered in their original direction. The exploratory HALP threshold for overall mortality was selected using the Youden index. Because the threshold was derived and evaluated in the same cohort, its selection was not included in the bootstrap internal-validation procedure and no claim of validated clinical performance was made. AUC differences between individual markers were not formally tested; comparisons therefore refer only to the observed AUC values.

To evaluate discrimination across clinically relevant horizons while accounting for censoring, cumulative/dynamic time-dependent ROC analyses were performed at 30 days, 90 days, 1 year, and 3 years. Cases were recipients who died by the specified horizon; controls were recipients event-free beyond that horizon. Inverse-probability-of-censoring weighting was used, and 95% confidence intervals were derived from the independent and identically distributed (iid) representation of the AUC estimator. These analyses were performed in R version 4.3.3 using the timeROC package version 0.4.1 [33,34,35]. Overall survival and the composite graft-related event-free endpoint were estimated by Kaplan–Meier analysis. Group differences were tested by log-rank, with Breslow and Tarone–Ware tests added as sensitivity analyses. Numbers at risk were displayed below the Kaplan–Meier curves. The median observed follow-up was calculated from transplantation to death or last clinical contact, and the median potential follow-up was estimated using the reverse Kaplan–Meier method [36].

Mortality-associated factors were examined by Cox proportional hazards regression. For clinical interpretability, the ROC-derived HALP group was used in Kaplan–Meier analyses and the main clinical Cox model, while continuous HALP was tested in sensitivity analyses. Covariates for the multivariable model were chosen from univariable *p* values together with clinical relevance and event count [37]. The base model included HALP, MELD score, and serum albumin. Because albumin is a component of HALP, this model was interpreted as a sensitivity analysis examining whether the composite HALP score provided prognostic information beyond albumin, not as evidence that HALP was completely independent of albumin. The incremental contribution of HALP to MELD was evaluated using the likelihood ratio test. For clinically interpretable scaling, hazard ratios for continuous HALP were additionally expressed per 0.1-unit increase.

We checked the proportional-hazards assumption with Schoenfeld residuals and log-minus-log plots. Because AST and ALT were strongly correlated (Spearman r = 0.84), they were never placed in the same model. Model discrimination was reported using Harrell’s C-index [38]; an optimism-corrected C-index and 95% confidence interval were calculated using 1000 bootstrap resamples. Firth penalized Cox regression was applied to assess the instability of standard Cox estimates for variables with few events, such as hypertension, and the apparently protective associations in these subgroups were not interpreted causally. All tests were two-sided, with *p* < 0.05 taken as significant. Secondary and exploratory analyses were interpreted as hypothesis-generating.

## 3. Results

### 3.1. Patient Characteristics

The final cohort comprised 194 recipients (Supplementary Figure S1; see Section 2.2 for the selection flow). Of these, 129 (66.5%) were men and 65 (33.5%) women, with a median age of 57 years (IQR 45–63; range 18–73). Living-donor transplantation was performed in 186 recipients (95.9%), whereas 8 (4.1%) received deceased-donor grafts. Diabetes mellitus was present in 42 recipients (21.6%), hypertension in 49 (25.3%), and coronary artery disease in 12 (6.2%). The median preoperative MELD score was 18 (IQR 14–23). The median observed follow-up was 1108 days (IQR 346–1938), while the median potential follow-up was 1544 days. During follow-up, 43 recipients (22.2%) died and 151 (77.8%) survived; composite graft-related events occurred in 44 recipients (22.7%). Table 1 presents the cohort characteristics.

### 3.2. Comparison Between Non-Survivors and Survivors

We compared non-survivors (n = 43) and survivors (n = 151) by Mann–Whitney U test. HALP was markedly lower in non-survivors than survivors [0.52 (IQR 0.39–0.67) vs. 0.77 (IQR 0.54–1.04); *p* < 0.001]. Hemoglobin was likewise lower in those who died [11.70 (IQR 10.48–12.25) g/dL vs. 12.20 (IQR 10.55–13.30) g/dL; *p* = 0.042]. Albumin was also lower among non-survivors [2.70 (IQR 2.40–3.10) g/dL vs. 3.10 (IQR 2.60–3.50) g/dL; *p* = 0.005]. Age, MELD, AST, ALT, lymphocyte count, platelet count, and the De Ritis ratio did not differ between groups (all *p* > 0.05). Among categorical variables, hypertension was more common in survivors than non-survivors (29.8% vs. 9.3%; *p* = 0.011).

Among the 43 deaths, 20 (47%) occurred within the first 30 days, four between days 31 and 90, and the remaining 19 beyond 90 days; overall, 24 deaths (56%) occurred within the first 90 days, with a median time to death of 32 days. In the low-HALP group, 16 of the 33 deaths (48%) occurred within the first 30 days. The causes of death were predominantly disease or tumor recurrence (including hepatocellular carcinoma recurrence), infection (including COVID-19/pneumonia), cardiovascular events (cardiogenic shock, myocardial infarction, or pulmonary embolism, and post-transplant heart failure), and multiorgan failure, whereas surgical or technical complications (hepatic artery–biliary fistula, post-procedural intrahepatic hemorrhage, and hemorrhagic shock) and hyperacute rejection together accounted for only a minority of deaths.

### 3.3. ROC Analysis and HALP Score Threshold

**Figure 1:**
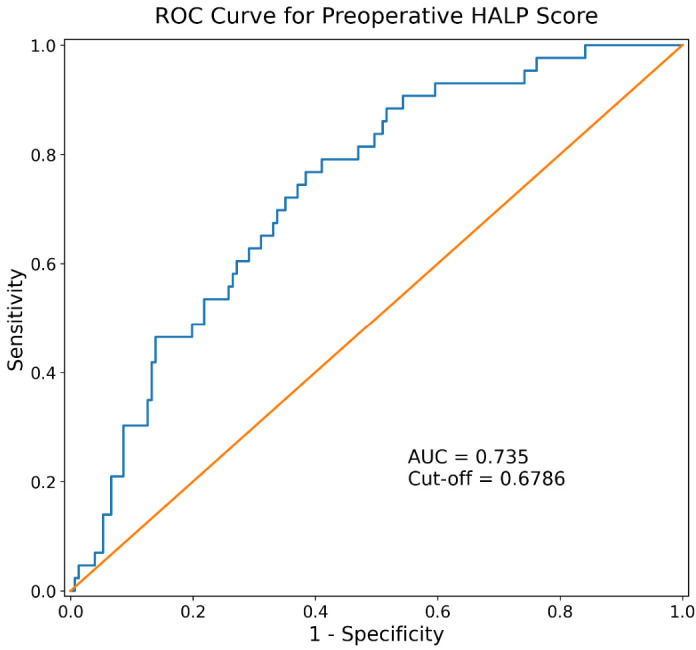
ROC curve for the HALP score. The area under the curve (AUC) was 0.735 (95% CI: 0.657–0.814; *p* < 0.001). The exploratory Youden-index threshold was 0.6786, with a sensitivity of 76.7% and a specificity of 61.6%.

Conventional ROC analysis for overall mortality yielded an AUC of 0.735 for HALP (95% CI 0.657–0.814; *p* < 0.001), which was the highest observed AUC among the evaluated markers (Figure 1). No formal pairwise comparison of AUCs was performed. The exploratory Youden-index threshold of 0.6786 provided 76.7% sensitivity and 61.6% specificity. Albumin (AUC 0.640; *p* = 0.005) and hemoglobin (AUC 0.602; *p* = 0.042) also showed significant discrimination. MELD (AUC 0.582; *p* = 0.102) and the De Ritis ratio (AUC 0.553; *p* = 0.291) did not significantly discriminate overall mortality (Table 2).

### 3.4. Distribution of HALP Groups

Using the determined threshold value of 0.6786, patients were divided into two categories, as follows: the low-HALP group (HALP < 0.6786; n = 91) and the high-HALP group (HALP ≥ 0.6786; n = 103). During follow-up, 33 patients (36.3%) in the low-HALP group and 10 patients (9.7%) in the high-HALP group died; this difference was statistically significant (χ^2^ = 19.748; *p* < 0.001).

### 3.5. Survival Analyses

#### 3.5.1. Overall Survival According to HALP Group

**Figure 2:**
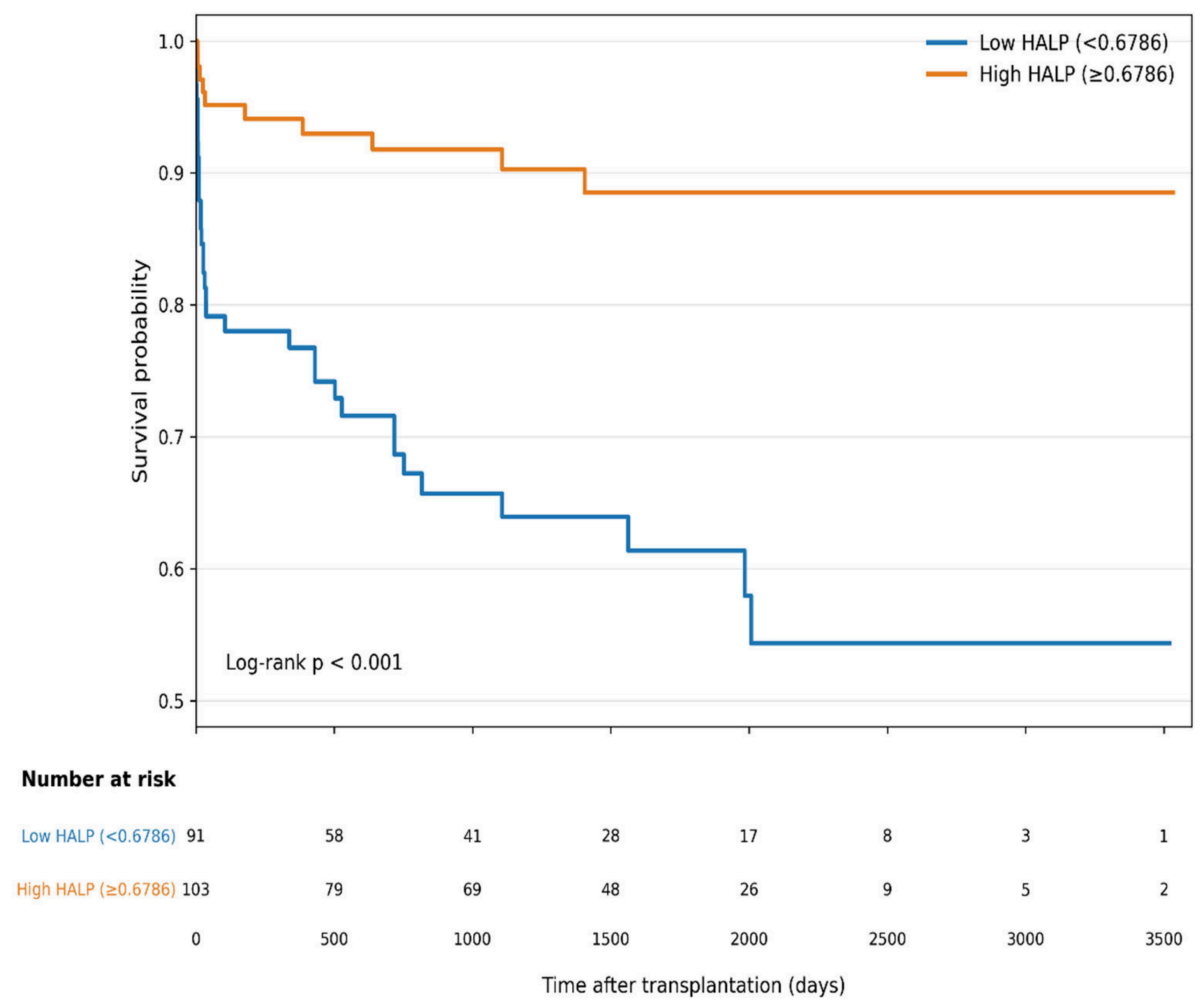
Kaplan–Meier overall survival curves according to HALP score groups, with numbers at risk. Survival differed between the high-HALP group (≥0.6786; n = 103) and low-HALP group (<0.6786; n = 91) (log-rank *p* < 0.001). Because the threshold was derived in the same cohort, these group-based estimates are exploratory and require external validation.

Kaplan–Meier analysis showed a significant difference in overall survival between the HALP groups (log-rank χ^2^ = 20.447; *p* < 0.001). Estimated mean survival was 3184.0 days in the high-HALP group versus 2189.7 days in the low-HALP group. The Breslow (*p* < 0.001) and Tarone–Ware (*p* < 0.001) tests agreed (Figure 2, Table 3).

#### 3.5.2. Survival According to Other Variables

Survival did not differ by sex (*p* = 0.875), donor type (*p* = 0.232), or etiology (*p* = 0.923). By De Ritis ratio, mean survival was 3326.8 days (low) versus 2649.8 days (high), a borderline difference (log-rank *p* = 0.065).

#### 3.5.3. Graft-Related Event-Free Survival

Composite graft-related events occurred in 44 patients (22.7%). In the Kaplan–Meier analysis according to HALP group, estimated mean graft-related event-free survival was 3184.0 days in the high-HALP group versus 2132.7 days in the low-HALP group (log-rank *p* < 0.001). However, because 43 of the 44 events coincided with death, this endpoint largely reflected overall survival. Therefore, in the present cohort, it should be interpreted as a secondary descriptive finding rather than as an independent graft-failure endpoint.

### 3.6. Cox Regression Analyses

#### 3.6.1. Univariable Analysis

On univariable Cox regression, the factors significantly associated with mortality were HALP group (HR 0.225; 95% CI 0.111–0.456; *p* < 0.001), continuous HALP (HR 0.783 per 0.1-unit increase; 95% CI 0.697–0.880; *p* < 0.001), albumin (HR 0.469; 95% CI 0.270–0.813; *p* = 0.007), MELD (HR 1.066; 95% CI 1.012–1.122; *p* = 0.016), and hypertension (HR 0.269; 95% CI 0.096–0.753; *p* = 0.012). The apparently protective hypertension association was based on only four deaths among hypertensive recipients and was considered vulnerable to sparse-data bias rather than a causal protective effect. This estimate is therefore unstable and is reported for completeness only; it should not be read as evidence that hypertension protects against post-transplant death. For this reason, hypertension was not entered into the main multivariable model, and its effect was examined only in a sensitivity analysis using Firth penalized Cox regression (Section 3.6.2). Exploratory Cox analyses showed statistically significant but clinically negligible associations for AST and ALT (AST: HR 1.003 per 10 U/L, 95% CI 1.001–1.005, *p* = 0.003; ALT: HR 1.005 per 10 U/L, 95% CI 1.002–1.009, *p* = 0.004). Because these markers did not differ between survivors and non-survivors, did not discriminate mortality in ROC analysis, and were markedly skewed with extreme values, they were not interpreted as clinically meaningful. All univariable results are summarized in Table 4.

#### 3.6.2. Multivariable Analysis

In the main model, high versus low HALP remained associated with lower mortality after adjustment for MELD and albumin (HR 0.239; 95% CI 0.116–0.493; *p* < 0.001). MELD also remained associated with mortality (HR 1.060; 95% CI 1.006–1.117; *p* = 0.030), whereas albumin was not significant in this joint model (HR 0.700; 95% CI 0.403–1.216; *p* = 0.205). Because albumin is a component of HALP, this analysis indicates that the composite HALP score provided additional prognostic information beyond albumin within this model; it does not establish complete independence from albumin. The model C-index was 0.737. Bootstrap internal validation of model discrimination yielded an optimism-corrected C-index of 0.723 (95% CI 0.642–0.803). This validation assessed the prespecified fitted models and did not repeat selection of the Youden threshold within each bootstrap sample. Adding HALP group to MELD improved model fit (likelihood-ratio χ*^2^* = 21.6; *p* < 0.001). When HALP was modeled continuously, the association persisted (HR 0.775 per 0.1-unit increase; 95% CI 0.686–0.877; *p* < 0.001). In the Firth penalized sensitivity analysis including hypertension, the HALP estimate was essentially unchanged (HR 0.235; 95% CI 0.114–0.485; *p* < 0.001), whereas the hypertension estimate remained based on sparse events and was not interpreted causally (Supplementary Table S2).

### 3.7. Additional Analyses

HALP did not differ across the ten etiology groups on Kruskal–Wallis testing (H = 9.405; *p* = 0.401). Living-donor (n = 186) and deceased-donor (n = 8) recipients were comparable for HALP (*p* = 0.313) and MELD (*p* = 0.098). This finding should be interpreted cautiously because of the small sample size of the deceased-donor group.

### 3.8. Discrimination Across Time Horizons

Cumulative/dynamic time-dependent ROC analysis accounting for censoring showed that HALP discriminated mortality consistently at 30 days (AUC 0.738; 95% CI 0.643–0.832; *p* < 0.001), 90 days (AUC 0.714; 95% CI 0.620–0.808; *p* < 0.001), 1 year (AUC 0.713; 95% CI 0.621–0.804; *p* < 0.001), and 3 years (AUC 0.729; 95% CI 0.643–0.815; *p* < 0.001). MELD showed significant discrimination at 90 days (AUC 0.643; 95% CI 0.523–0.762; *p* = 0.019) and 1 year (AUC 0.658; 95% CI 0.547–0.768; *p* = 0.005), but not at 30 days (AUC 0.616; 95% CI 0.481–0.752; *p* = 0.092) or 3 years (AUC 0.573; 95% CI 0.459–0.687; *p* = 0.209). The De Ritis ratio did not show significant discrimination at any evaluated horizon (Supplementary Table S1).

## 4. Discussion

In this single-center retrospective cohort of 194 adults transplanted for non-malignant disease, lower preoperative HALP was associated with higher all-cause post-transplant mortality. Mortality occurred in 36.3% of the low-HALP group and 9.7% of the high-HALP group. HALP had the highest observed AUC among the evaluated markers in conventional ROC analysis for overall mortality (AUC 0.735), although pairwise AUC differences were not formally tested. The high-versus-low HALP association persisted after adjustment for MELD and albumin (HR 0.239; 95% CI 0.116–0.493), and addition of HALP group improved model fit. These findings support HALP as a potentially complementary, rather than competing, pretransplant risk marker that requires external validation.

Accurate prediction of survival after liver transplantation is clinically important for patient counseling, perioperative planning, and the efficient use of limited donor resources. Although traditional scoring systems are strong indicators of disease severity, they assess the physiological reserve related to host biology only to a limited extent [9,10]. End-stage liver disease is a chronic systemic condition characterized by sarcopenia, malnutrition, immune dysregulation, and cytopenias related to portal hypertension, and the recipient’s ability to tolerate this chronic stress plays an independent role in post-transplant outcomes [7,8]. Therefore, simple and objective markers that reflect host biological reserve and complement MELD are needed.

MELD and HALP appeared to capture partly different prognostic domains. MELD remained associated with mortality in the Cox model, whereas its conventional AUC for overall mortality was not significant. Cumulative/dynamic time-dependent ROC analysis clarified this apparent discrepancy, as follows: MELD discriminated mortality at 90 days and 1 year but not at 30 days or 3 years, while HALP showed consistent discrimination across all four horizons. These results are compatible, with MELD primarily reflecting short-term hepatorenal disease severity and HALP potentially reflecting broader host reserve. Nevertheless, no formal pairwise comparison between HALP and MELD AUCs was performed, so the data should not be interpreted as demonstrating statistical superiority of HALP.

The HALP score’s prognostic value rests on a biologically plausible basis. Its components (hemoglobin, albumin, lymphocytes, and platelets) provide indirect information on oxygen-carrying capacity and chronic disease burden, hepatic synthesis and nutritional reserve, cellular immune competence, and thrombo-inflammatory activity, respectively [14,15,24]. In liver transplant candidates, all of these domains may be affected by the disease process and may be related to surgical stress, immunosuppression, infection risk, and recovery capacity [5,6,13]. Therefore, HALP can be interpreted not as a single laboratory parameter, but as a practical reserve marker that summarizes multiple physiological domains.

Albumin was associated with mortality in univariable analysis but was not significant after HALP and MELD were entered in the same model. Because albumin is one of the four components of HALP, the joint model evaluates whether the composite HALP score contributes prognostic information beyond albumin; it cannot demonstrate that HALP is completely independent of albumin. The preserved continuous-HALP association (HR 0.775 per 0.1-unit increase; 95% CI 0.686–0.877) suggests that the finding was not solely dependent on the exploratory dichotomization, but the shared component structure and potential collinearity warrant cautious interpretation.

Our findings agree with earlier reports linking low HALP scores to poorer survival and higher mortality across diverse malignant and non-malignant conditions [14,16,17,18,19,20,21,22,23,24]. In liver-related diseases, low HALP scores have also been associated with poor prognosis in hepatobiliary malignancies [25] and with advanced disease stage and increased mortality risk in chronic liver diseases [29,30]. In the context of liver transplantation, however, existing data are largely limited to hepatocellular carcinoma cohorts. A recent two-center study found low preoperative HALP independently related to overall survival but not recurrence-free survival [31], and another series tied HALP to early post-transplant outcomes [32]. Because much of this literature is based on malignancy-related cohorts, tumor burden, recurrence risk, and cancer-related systemic inflammation may confound the prognostic role of HALP as a host-related marker. Our study adds to this by testing HALP in a non-malignant transplant population free of tumor-related confounders. That HALP related to overall but not recurrence-free survival in a malignant cohort further supports its reading as a host-reserve marker rather than a tumor-biology marker [31].

Although worse graft-event-free survival was observed in the low-HALP group, this finding should be interpreted cautiously because graft-loss events almost completely overlapped with death. Therefore, death-censored graft survival and competing-risk analysis could not be meaningfully evaluated in this cohort. The present data do not show that HALP independently predicts graft failure; rather, they indicate that low HALP marks the overall burden of poor post-transplant outcomes. Larger cohorts with a higher number of events are needed to more accurately evaluate outcomes such as graft loss independent of death and retransplantation.

The De Ritis ratio did not significantly discriminate mortality in this cohort and did not show independent prognostic value in Cox regression analyses. This may suggest that the ratio has a limited role in post-transplant prognosis in non-malignant transplant populations, as it is more closely related to patterns of acute liver injury. Exploratory univariable Cox models suggested statistically significant but very small positive associations for AST and ALT; however, these markers did not differ significantly between survivors and non-survivors and did not discriminate mortality in ROC analysis. Given their markedly skewed distributions and extreme outliers, these findings were considered unstable and were not interpreted as clinically meaningful. The apparently lower mortality associated with hypertension in univariable analysis should also not be considered a true protective effect. The small number of events in this subgroup makes the association vulnerable to sparse-data bias [37]. In the Firth penalized Cox sensitivity analysis, the HALP association remained essentially unchanged, supporting its robustness to inclusion of this unstable subgroup term [39].

From a clinical perspective, the main advantage of the HALP score is its applicability. Unlike imaging-based sarcopenia measurements, which require additional cost and technical resources, or subjective global assessment, which is open to observer-related variability, HALP is entirely objective and can be calculated without additional cost from four blood parameters routinely measured in clinical practice [5]. In this respect, HALP should not be considered a replacement for performance-based frailty measures, but rather as a practical laboratory-based screening indicator that may help identify high-risk patients who require more detailed assessment [6,11,12,13]. In particular, a low HALP value can help flag patients who require more comprehensive evaluation in terms of nutritional status, anemia, inflammation, and overall frailty during the pretransplant period, and may also help target prehabilitation and nutritional optimization strategies [40,41,42]. However, the present study does not show that interventions targeting HALP components improve transplant outcomes. Therefore, HALP should be regarded not as a tool that independently guides clinical decisions, but as an auxiliary marker that may contribute to risk assessment.

Two consequences follow for practice and for research. In practice, a low preoperative HALP value is best treated as a prompt rather than a decision rule, as follows: it identifies a recipient in whom nutritional status, anemia, chronic inflammation, and physical frailty deserve structured assessment before transplantation, using the established instruments for each domain rather than the score itself [5,6]. HALP should not influence candidacy or allocation. In research, the open question is whether the reserve that HALP reflects is modifiable. The present observational design cannot answer this, as follows: an association between a preoperative laboratory value and later mortality does not establish that raising that value improves outcomes, and the components of the score are themselves downstream markers of disease severity. Answering it requires prospective studies in which candidates with a prespecified low HALP value are assigned to structured prehabilitation, including protein-targeted nutritional support, supervised exercise, and correction of anemia and of treatable sources of inflammation, with post-transplant survival and complications as endpoints and with HALP measured serially rather than only at baseline [40,41,42]. Multicenter designs would also allow the exploratory threshold reported here to be validated, or replaced, in an independent population.

Several limitations frame these results. The single-center retrospective design leaves potential for selection bias and residual confounding. Important variables such as immunosuppressive protocols, donor characteristics, ischemia times, perioperative complications, post-transplant infections, and detailed nutritional interventions were unavailable. The great majority of our recipients received a living-donor graft (186/194, 95.9%), with only 8 (4.1%) receiving a deceased-donor graft. Living- and deceased-donor recipients may differ in perioperative risk, graft characteristics, and early post-operative course; this marked predominance of living-donor transplantation therefore represents a potential confounding factor and may limit the generalizability of our results. The small number of deceased-donor recipients precluded a meaningful donor-type subgroup analysis. The HALP threshold of 0.6786 was selected and tested in the same cohort; threshold selection was not repeated within the bootstrap internal-validation procedure. Consequently, the group-based effect estimates may be optimistic and the threshold must not be treated as a validated clinical cut-off. The limited number of deaths constrained model complexity, and HALP was measured at only one pretransplant time point. External multicenter validation, preferably with prespecified thresholds and direct comparison with frailty and sarcopenia measures, is required.

## 5. Conclusions

In this cohort, lower preoperative HALP was associated with higher post-transplant mortality among adults undergoing liver transplantation for non-malignant indications. HALP showed consistent time-dependent discrimination across the evaluated horizons and provided additional model information when considered with MELD and albumin. These findings support further evaluation of HALP as an easily calculated complementary risk marker, but they do not validate the cohort-derived threshold or establish that HALP-guided interventions improve outcomes. Prospective multicenter studies should externally validate the continuous association and any proposed threshold before clinical implementation.

## Figures and Tables

**Table 1:** Demographic and clinical characteristics of the patients and comparison between non-survivors and survivors.

Variable	All Patients (n = 194)	Non-Survivors (n = 43)	Survivors (n = 151)	*p*
**Continuous variables—Median (IQR)**				
Age (years)	57 (46–62)	59 (50–63)	57 (45–61)	0.191
MELD score	18 (15–24)	21 (16–24)	18 (15–24)	0.101
AST (U/L)	52 (33–77)	55 (38–71)	50 (32–78)	0.574
ALT (U/L)	32 (22–54)	32 (22–57)	32 (23–54)	0.978
Hemoglobin (g/dL)	12.08 (10.53–12.90)	11.70 (10.48–12.25)	12.20 (10.55–13.30)	**0.042**
Albumin (g/dL)	3.00 (2.60–3.50)	2.70 (2.40–3.10)	3.10 (2.60–3.50)	**0.005**
Lymphocyte count (/µL)	1855 (1373–2488)	1550 (1285–2325)	1900 (1475–2585)	0.090
Platelet count (/µL)	91,550 (68,900–125,000)	113,000 (77,250–132,500)	89,500 (67,750–119,500)	0.090
De Ritis ratio (AST/ALT)	1.50 (1.22–1.91)	1.61 (1.23–2.01)	1.48 (1.22–1.89)	0.292
HALP score	0.696 (0.493–0.978)	0.518 (0.392–0.672)	0.773 (0.539–1.040)	**<0.001**
**Categorical variables—n (%)**				
Sex				0.881
Male	129 (66.5)	29 (67.4)	100 (66.2)	
Female	65 (33.5)	14 (32.6)	51 (33.8)	
Donor type				0.074 ^a^
Living donor	186 (95.9)	39 (90.7)	147 (97.4)	
Deceased donor	8 (4.1)	4 (9.3)	4 (2.6)	
Diabetes mellitus	42 (21.6)	5 (11.6)	37 (24.5)	0.092 ^a^
Hypertension	49 (25.3)	4 (9.3)	45 (29.8)	**0.005 ^a^**
Coronary artery disease	12 (6.2)	5 (11.6)	7 (4.6)	0.143 ^a^
HALP				**<0.001**
Low (<0.6786)	91 (46.9)	33 (76.7)	58 (38.4)	
High (≥0.6786)	103 (53.1)	10 (23.3)	93 (61.6)	

Continuous variables are presented as median (IQR) and were compared using the Mann–Whitney U test. Categorical variables are presented as n (%) and were compared using the Pearson chi-square test. ^a^ Fisher’s exact test. Significant *p* values are shown in bold (*p* < 0.05). IQR: interquartile range; MELD: Model for End-stage Liver Disease; AST: aspartate aminotransferase; ALT: alanine aminotransferase; HALP: Hemoglobin–Albumin–Lymphocyte–Platelet.

**Table 2:** ROC curve analysis results for predicting mortality.

Variable	AUC	Std. Error	95% CI	*p*	Threshold	Sensitivity (%)	Specificity (%)
**HALP Score**	**0.735**	0.040	**0.657–0.814**	**<0.001**	0.6786	76.7	61.6
Albumin (g/dL)	0.640	0.050	0.542–0.738	0.005	—	—	—
Hemoglobin (g/dL)	0.602	0.044	0.515–0.688	0.042	—	—	—
Lymphocyte count (/µL)	0.585	0.050	0.486–0.683	0.090	—	—	—
De Ritis ratio	0.553	0.050	0.455–0.651	0.291	—	—	—
MELD score	0.582	0.050	0.483–0.681	0.102	—	—	—
AST (U/L)	0.472	0.049	0.375–0.568	0.573	—	—	—
ALT (U/L)	0.501	0.050	0.403–0.600	0.977	—	—	—
Platelet count (/µL)	0.415	0.048	0.322–0.508	0.090	—	—	—

The threshold value was calculated only for the HALP score using the Youden index. AUC: area under the curve; CI: confidence interval; HALP: Hemoglobin–Albumin–Lymphocyte–Platelet; MELD: Model for End-stage Liver Disease.

**Table 3:** Kaplan–Meier survival analysis: mean survival times according to HALP group and other variables.

Variable	Group	n	Events	Mean Survival (days)	95% CI	Log-Rank *p*
**HALP**	Low HALP (<0.6786)	91	33	2189.7	1833.7–2545.8	**<0.001**
	High HALP (≥0.6786)	103	10	3184.0	2978.5–3389.5	
Sex	Male	129	29	2705.6	2440.6–2970.5	0.875
	Female	65	14	2736.1	2373.5–3098.6	
Donor type	Living donor	186	39	2767.3	2553.1–2981.4	0.232
	Deceased donor	8	4	2200.0	1491.6–2908.5	
De Ritis group	Low (<1.0)	18	1	3326.8	2986.6–3666.9	0.065
	High (≥1.0)	176	42	2649.8	2417.6–2882.1	
Etiology	10 groups	194	43	—	—	0.923
**Graft-related event-free survival (HALP)**	Low HALP (<0.6786)	91	34 ^a^	2132.7	1773.4–2492.0	**<0.001**
	High HALP (≥0.6786)	103	10 ^a^	3184.0	2978.5–3389.5	

Survival estimates were restricted to the longest survival time when the longest observation was censored. ^a^ Composite graft-related event (total n = 44). CI: confidence interval; HALP: Hemoglobin–Albumin–Lymphocyte–Platelet.

**Table 4:** Univariable and multivariable Cox regression analysis results for mortality.

Variable	Univariable Analysis			Multivariable Analysis		
	HR	95% CI	*p*	HR	95% CI	*p*
Age (years)	1.019	0.989–1.049	0.212	—	—	—
**MELD score**	1.066	1.012–1.122	**0.016**	**1.060**	**1.006–1.117**	**0.030**
**AST (per 10 U/L) ^a^**	1.003	1.001–1.005	**0.003**	—	—	—
**ALT (per 10 U/L) ^a^**	1.005	1.002–1.009	**0.004**	—	—	—
Hemoglobin (g/dL)	0.870	0.737–1.026	0.098	—	—	—
Albumin (g/dL)	0.469	0.270–0.813	**0.007**	**0.700**	**0.403–1.216**	**0.205**
Lymphocyte count (per 100/µL)	0.974	0.938–1.011	0.168	—	—	—
Platelet count (per 10^4^/µL)	1.018	0.970–1.069	0.466	—	—	—
**De Ritis ratio (continuous)**	1.545	0.865–2.760	0.141	—	—	—
**De Ritis ratio (≥1.0)**	5.289	0.727–38.449	0.100	—	—	—
**HALP group** **(High vs. Low)**	**0.225**	**0.111–0.456**	**<0.001**	**0.239**	**0.116–0.493**	**<0.001**
**HALP score (per 0.1-unit increase)**	**0.783**	**0.697–0.880**	**<0.001**	—	—	—
Sex (Female vs. Male)	0.950	0.502–1.799	0.875	—	—	—
Donor type (deceased vs. living)	1.856	0.661–5.213	0.240	—	—	—
Diabetes mellitus (Yes vs. No)	0.451	0.178–1.147	0.095	—	—	—
**Hypertension** **(Yes vs. No)**	0.269	0.096–0.753	**0.012**	—	—	—
Coronary artery disease	2.088	0.821–5.310	0.122	—	—	—

The multivariable model included HALP group, MELD score, and serum albumin. Hypertension was not included in the main model because of sparse events; its inclusion in sensitivity analysis did not materially change the HALP estimate (HR ≈ 0.23). ^a^ AST and ALT HRs are reported per 10-U/L increase after rescaling coefficients from models entered in U/L; these variables were not included in the multivariable model because of high intercorrelation, marked skewness, extreme outliers, and non-significant ROC discrimination. The model C-index was 0.737. Significant *p* values are shown in bold. HR: hazard ratio; CI: confidence interval.

## Data Availability

The data presented in this study are available on reasonable request from the corresponding author. The data are not publicly available due to privacy and ethical restrictions.

## Supplementary Materials

The following supporting information can be downloaded at: https://www.mdpi.com/article/10.3390/jcm15155970/s1, Supplementary Table S1: Marker distributions and censoring-adjusted time-dependent discrimination of MELD, HALP, and the De Ritis ratio for post-transplant mortality; Supplementary Table S2: Cox Regression, Internal Validation, and Penalized Sensitivity Analysis for Post-Transplant Mortality; Supplementary Figure S1: Patient flow diagram.
