# Supplementary material for: Prognostic Value of the Preoperative HALP Score for Survival in Non-Malignant Liver Transplant Recipients
Source: J Clin Med. 2026 Jul 30;15(15):5970. doi: 10.3390/jcm15155970 (PMC13466314; doi:10.3390/jcm15155970)
Supplement: Supplementary file 1 [file jcm-15-05970-s001.zip › jcm-4420703-supplementary.pdf]

Supplementary Table S1. Marker distributions and censoring-adjusted time-dependent discrimination of MELD, HALP, and the De Ritis ratio for post-transplant mortality

A. Marker distributions by outcome status at each time horizon (descriptive)

Endpoint	Events, n	Event (+): marker, median (IQR)	Event (−): marker, median (IQR)	Welch t-test p	MWU p	Unadjusted AUC (95% CI)
			MELD score			
30-day mortality	20	21.0 (16.0–25.0)	18.0 (15.0–24.0)	0.075	0.088	0.616 (0.478–0.755)
90-day mortality	24	22.0 (16.0–25.0)	17.7 (14.6–24.0)	**0.018**	**0.019**	**0.647 (0.526–0.768)**
1-year mortality	27	23.0 (16.5–24.0)	17.4 (14.3–24.0)	**0.010**	**0.010**	**0.653 (0.542–0.764)**
3-year mortality	37	21.0 (16.0–24.0)	18.0 (15.0–24.0)	0.056	0.086	0.591 (0.485–0.696)
Overall mortality	43	21.0 (16.0–24.0)	18.0 (14.8–24.0)	0.069	0.101	0.582 (0.482–0.681)
			HALP score			
30-day mortality	20	0.53 (0.38–0.66)	0.72 (0.51–1.02)	**<0.001**	**<0.001**	**0.738 (0.642–0.833)**
90-day mortality	24	0.57 (0.39–0.66)	0.73 (0.51–1.02)	**<0.001**	**<0.001**	**0.715 (0.621–0.809)**
1-year mortality	27	0.57 (0.38–0.67)	0.74 (0.52–1.02)	**<0.001**	**<0.001**	**0.715 (0.624–0.805)**
3-year mortality	37	0.52 (0.40–0.67)	0.76 (0.53–1.04)	**<0.001**	**<0.001**	**0.726 (0.646–0.807)**
Overall mortality	43	0.52 (0.39–0.67)	0.77 (0.54–1.04)	**<0.001**	**<0.001**	**0.735 (0.657–0.814)**
			De Ritis ratio (AST/ALT)			
30-day mortality	20	1.65 (1.17–2.05)	1.48 (1.23–1.89)	0.236	0.273	0.575 (0.430–0.720)
90-day mortality	24	1.70 (1.17–2.05)	1.47 (1.23–1.88)	0.118	0.141	0.593 (0.460–0.726)
1-year mortality	27	1.68 (1.22–2.04)	1.48 (1.22–1.88)	0.143	0.176	0.581 (0.460–0.703)
3-year mortality	37	1.61 (1.24–2.03)	1.48 (1.21–1.89)	0.302	0.368	0.548 (0.442–0.653)
Overall mortality	43	1.61 (1.23–2.01)	1.48 (1.22–1.89)	0.212	0.292	0.553 (0.454–0.652)

Event (+) denotes recipients who died within the specified time window; Event (−) denotes all remaining recipients, including survivors and recipients who died after that window. For overall mortality, Event (+) denotes all non-survivors and Event (−) denotes survivors. Marker values are median (interquartile range). The unadjusted AUC is the conventional area under the ROC curve for this fixed binary classification; all unadjusted AUCs and their confidence intervals were calculated with the same nonparametric (DeLong) method. Because recipients are classified by observed status at a fixed time point, this panel does not account for censoring and is presented for descriptive comparison only. The censoring-adjusted estimates in panel B are the primary discrimination results and are the values reported in the main text. For HALP, the score direction was reversed so that higher analysis values denoted higher risk. Bold denotes $p < 0.05$ or an AUC confidence interval excluding 0.5. n = 194 with 43 deaths during follow-up. AUC: area under the receiver operating characteristic curve; CI: confidence interval; IQR: interquartile range; MWU: Mann–Whitney U test.

B. Cumulative/dynamic time-dependent ROC analysis (censoring-adjusted)

Marker	Direction of risk	30-day AUC (95% CI)	90-day AUC (95% CI)	1-year AUC (95% CI)	3-year AUC (95% CI)
MELD score	Higher values = higher risk	0.616 (0.481–0.752)	0.643 (0.523–0.762)	0.658 (0.547–0.768)	0.573 (0.459–0.687)
**HALP score**	Lower values = higher risk	**0.738 (0.643–0.832)**	**0.714 (0.620–0.808)**	**0.713 (0.621–0.804)**	**0.729 (0.643–0.815)**
De Ritis ratio	Higher values = higher risk	0.575 (0.433–0.717)	0.595 (0.464–0.725)	0.592 (0.472–0.711)	0.556 (0.446–0.666)

AUCs were estimated using cumulative/dynamic time-dependent ROC analysis. Cases were recipients who died by the specified horizon and controls were recipients who remained event-free beyond that horizon. Censoring was handled using inverse-probability-of-censoring weighting, and confidence intervals were derived from the iid representation of the AUC estimator. For HALP, the score direction was reversed so that higher analysis values consistently denoted higher risk. AUC: area under the curve; CI: confidence interval.

Interpretation

HALP showed consistent discrimination at all evaluated horizons. MELD showed significant discrimination at 90 days and 1 year, but not at 30 days or 3 years. The De Ritis ratio did not show significant discrimination at any horizon. These observed AUC patterns were not subjected to formal pairwise marker comparisons.

Supplementary Table S2. Cox Regression, Internal Validation, and Penalized Sensitivity Analysis for Post-Transplant Mortality

A. Cox proportional hazards regression

Variable	Univariable HR (95% CI)	p-value	Multivariable† HR (95% CI)	p-value
**HALP score (per 0.1-unit increase)**	**0.783 (0.697–0.880)**	**<0.001**	—	—
**HALP group (≥ 0.6786 vs < 0.6786)**	0.225 (0.111–0.456)	**<0.001**	0.239 (0.116–0.493)	**<0.001**
**MELD score**	1.066 (1.012–1.122)	**0.016**	1.060 (1.006–1.117)	**0.030**
**Albumin (g/dL)**	0.469 (0.270–0.813)	**0.007**	0.700 (0.403–1.216)	0.205
Age (years)	1.019 (0.989–1.049)	0.212	—	—
Sex (Female vs. Male)	0.950 (0.502–1.799)	0.875	—	—

† Multivariable model: HALP group, MELD score, and serum albumin. Because albumin is a component of HALP, this was interpreted as a sensitivity model assessing prognostic information beyond albumin, not complete independence from albumin. The continuous-HALP sensitivity model yielded HR 0.775 per 0.1-unit increase (95% CI 0.686–0.877; $p < 0.001$).

B. Discrimination (Harrell C-index) with bootstrap internal validation

Model	Apparent C-index	Optimism	Optimism-corrected C-index (95% CI)
**HALP group + MELD + albumin**	0.737	0.014	**0.723 (0.642–0.803)**
HALP score alone (continuous)	0.703	0.000	0.703 (0.629–0.777)
MELD score alone	0.595	0.003	0.592 (0.505–0.679)

Optimism was estimated using 1000 bootstrap resamples. This procedure internally validated discrimination of the fitted models but did not repeat selection of the Youden-derived HALP threshold within each bootstrap sample; it therefore does not validate the threshold of 0.6786.

C. Firth penalized Cox regression including hypertension (sparse-event sensitivity analysis)

Variable	HR (95% CI)	p-value
**HALP group (≥ 0.6786 vs < 0.6786)**	0.235 (0.114–0.485)	**<0.001**
**MELD score**	1.066 (1.012–1.123)	**0.015**
Albumin (g/dL)	0.750 (0.432–1.304)	0.309
Hypertension (present vs absent)	0.277 (0.103–0.743)	0.011

Firth penalized likelihood was used because hypertension was associated with very few events (4 deaths among hypertensive patients), which can bias standard Cox estimates. The HALP group effect was essentially unchanged versus the main model, indicating robustness. The apparently protective association of hypertension is based on sparse data and was not interpreted as causal.

Note: A competing-risk analysis for graft loss was not performed because graft loss and death were almost entirely concordant in this cohort (43 of 44 graft-loss events coincided with death; no graft-independent deaths occurred), so no competing event was available.

Supplementary Figure S1. Patient flow diagram

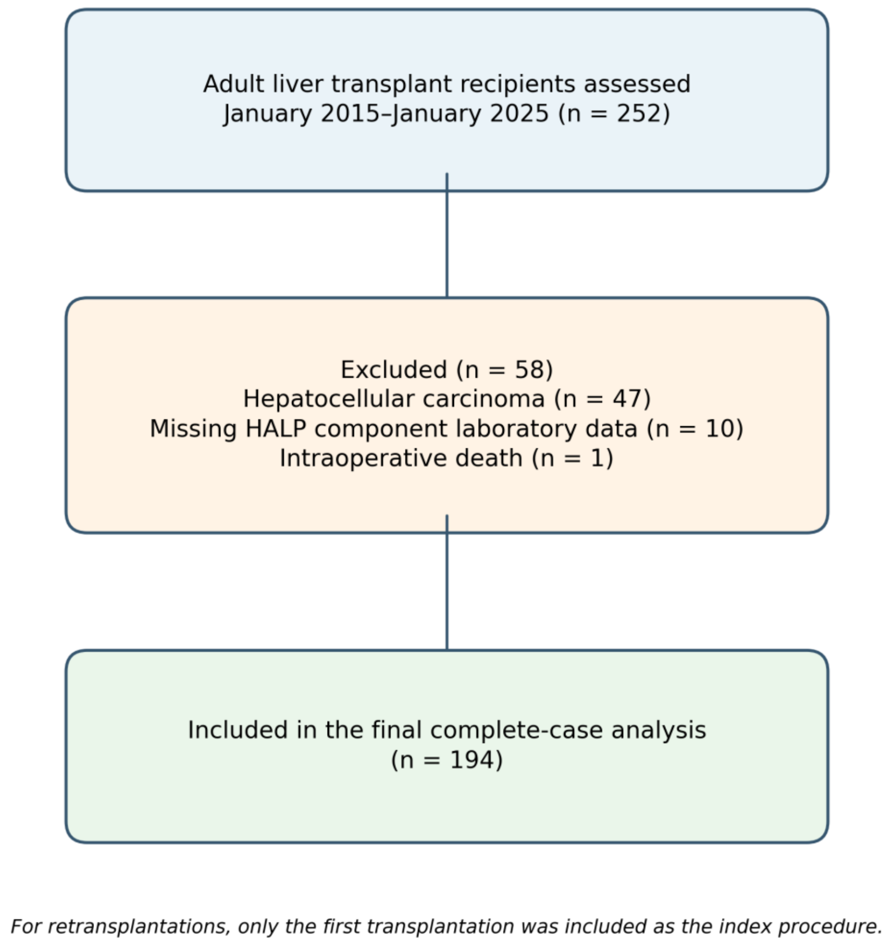

Supplementary Figure S1. Flow of adult liver transplant recipients through cohort selection. HALP: Hemoglobin–Albumin–Lymphocyte–Platelet.
